# Heterocyclic Chemistry Applied to the Design of *N*-Acyl Homoserine Lactone Analogues as Bacterial Quorum Sensing Signals Mimics

**DOI:** 10.3390/molecules26175135

**Published:** 2021-08-24

**Authors:** Qiang Zhang, Sizhe Li, Maha Hachicha, Mohamed Boukraa, Laurent Soulère, Mohamed L. Efrit, Yves Queneau

**Affiliations:** 1 Univ Lyon, INSA Lyon, Université Claude Bernard Lyon 1, CPE Lyon, UMR 5246, CNRS, ICBMS, Institut de Chimie et de Biochimie Moléculaires et Supramoléculaires, Bât. E. Lederer, 1 Rue Victor Grignard, F-69622 Villeurbanne, France; zerq123@163.com (Q.Z.); s.li@lic.leidenuniv.nl (S.L.); hachicha19@gmail.com (M.H.); mohamed.boukraa@fst.utm.tn (M.B.); 2 Laboratoire de Synthèse Organique Sélective et Hétérocyclique, Faculté des Sciences de Tunis, Université de Tunis El Manar, El Manar, Tunis 2092, Tunisia

**Keywords:** acyl homoserine lactones, AHL, quorum sensing, bioisosteres, heterocyclic chemistry

## Abstract

*N*-acyl homoserine lactones (AHLs) are small signaling molecules used by many Gram-negative bacteria for coordinating their behavior as a function of their population density. This process, based on the biosynthesis and the sensing of such molecular signals, and referred to as Quorum Sensing (QS), regulates various gene expressions, including growth, virulence, biofilms formation, and toxin production. Considering the role of QS in bacterial pathogenicity, its modulation appears as a possible complementary approach in antibacterial strategies. Analogues and mimics of AHLs are therefore biologically relevant targets, including several families in which heterocyclic chemistry provides a strategic contribution in the molecular design and the synthetic approach. AHLs consist of three main sections, the homoserine lactone ring, the central amide group, and the side chain, which can vary in length and level of oxygenation. The purpose of this review is to summarize the contribution of heterocyclic chemistry in the design of AHLs analogues, insisting on the way heterocyclic building blocks can serve as replacements of the lactone moiety, as a bioisostere for the amide group, or as an additional pattern appended to the side chain. A few non-AHL-related heterocyclic compounds with AHL-like QS activity are also mentioned.

## 1. Introduction

### 1.1. The Power of Heterocyclic Chemistry for the Design of Bioactive Molecules

Heterocyclic chemistry represents an essential component of organic chemistry, offering high-performance synthetic tools searching for biologically active molecules. In recent decades, this chemistry has experienced considerable growth both in terms of the number of publications and of biological applications. Indeed, heterocycles, whether synthetic or natural, are important, not only for their abundance and extraordinary diversity, but above all for their biological, medicinal, and therapeutic potential. Thus, elaborate architectures containing one or more heterocyclic nuclei have been either synthesized or extracted from natural resources.

**Figure 1:**
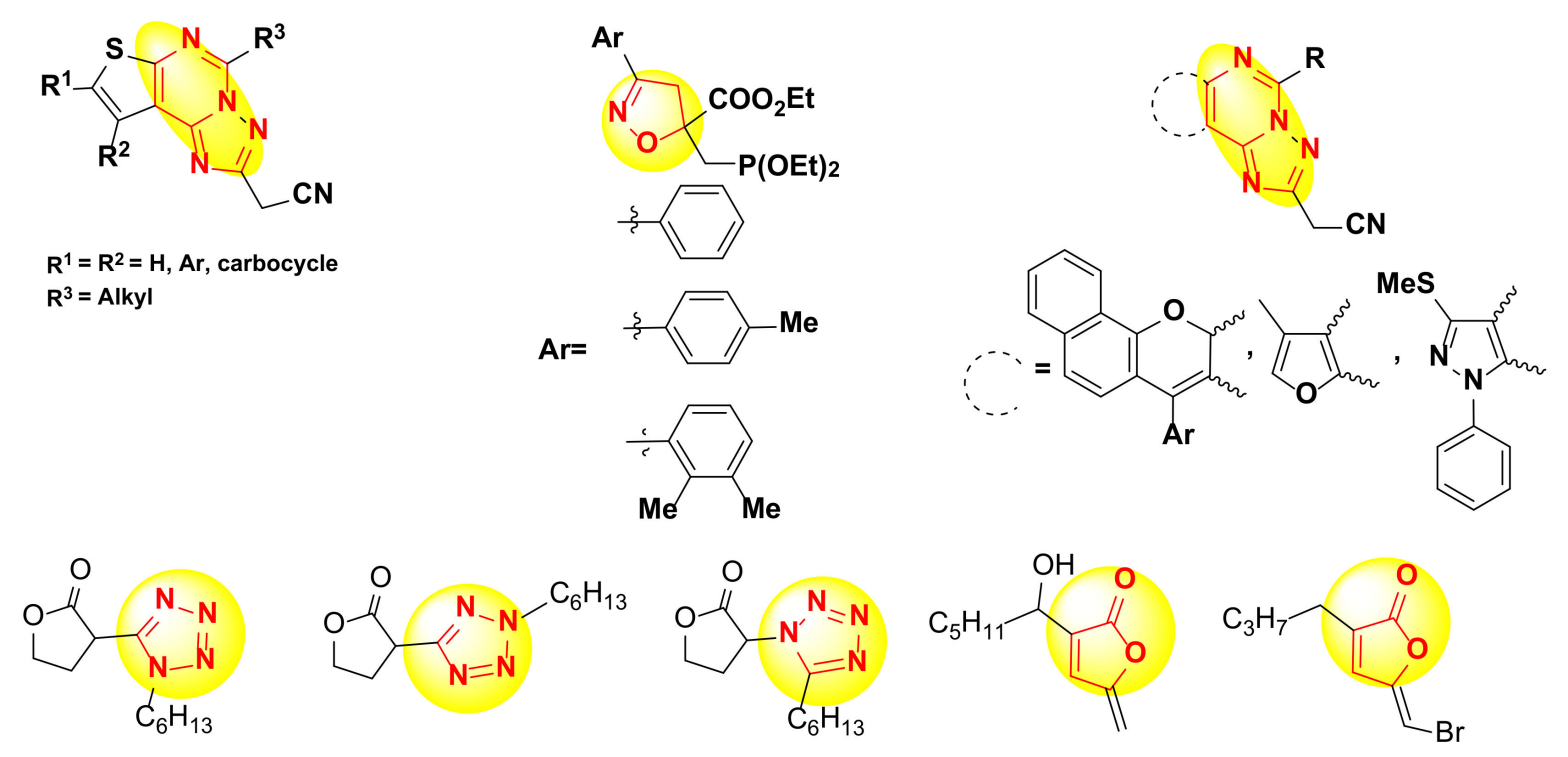
Several families of biologically relevant heterocyclic compounds, such as condensed pyrimidines, isoxazolines, pyrazolines, tetrazole, and furanone derivatives.

The pharmacological applications of these heterocycles vary, in particular as anxiolytic [1], anti-bacterial [2], anti-fungal [3,4], anti-inflammatory [5], anti-thrombotic [6], analgesic [7], antitumor [8,9,10], etc. Our research teams have specialized in the synthesis of several families of new biologically relevant heterocyclic compounds. Recent works have shown that condensed pyrimidines, isoxazolines, and pyrazolines exhibit significant antibacterial activity [11,12,13]. Other studies were devoted to the development of heterocyclic systems as quorum sensing modulators, including tetrazole and furanone derivatives (Figure 1) [14,15]. 

When designing molecules endowed with biological activity, it has been noted that certain heterocyclic fragments, of different structure, called bioisosteres, are recognized in a similar way by biological systems. The role of these bioisosteres is to mimic certain patterns present within the biomolecules.

However, the introduction of these bioisosteres frequently generates structural changes, which may be beneficial or harmful depending on the context, in relation to size, geometry, electronic distribution, polarizability, dipole, polarity, lipophilicity and pKa, which play a key role in molecular recognition and mimicry [16]. Recall that Hansch et al. [17] were the first to establish the relationship between physicochemical properties and biological activity. This has led to the discovery of a panoply of new biomolecules whose basic structures have already been proven, and/or have even made it possible to improve their therapeutic properties.

The introduction of a heterocyclic moiety into the active substance is important in multiple ways. Indeed, the binding of these compounds to receptors induces a change in conformation the therapeutic target [16] due to (a) the geometry of the heterocycle; (b) following electrostatic-type interactions; and (c) by hydrogen bonding. 

For these different interactions to take place, a drug–receptor affinity is necessary. Biologists, with the help of chemists, are increasingly working to improve both the selectivity and the specificity of these heterocycles towards the target [18].

The purpose of this review is to give the state of the art focusing on the use of heterocyclic cores in the design of analogues of *N*-acyl homoserine lactones (AHLs), which are small molecular signaling molecules used by many Gram-negative bacteria for coordinating their behavior as a function of their population density in a process referred to as Quorum Sensing (QS).

### 1.2. Role of Acyl Homoserine Lactones in Bacterial Quorum Sensing

Bacterial quorum sensing (QS) is a cell-cell communication pathway used by bacteria to coordinate their population density. Bacteria, though being monocellular organisms, are capable, at high population density and thanks to the QS signaling process, of behaving like multicellular entities. Quorum sensing is used by many pathogenic bacteria to regulate the transcription of specific genes, and consequently the expression of specific phenotypes, among which biofilm formation, virulence, growth, and bioluminescence [19,20]. It relies on the production, diffusion, and detection of small molecular chemical signals referred to as autoinducers. When the autoinducers produced by a synthase in each cell accumulate and reach a critical concentration, they can be detected by their cognate receptor and then activate gene transcription [21,22,23]. Chemical modulation of QS provides an opportunity to interfere with virulence, and possibly contribute to new anti-infection strategies.

Among the different types of autoinducers, acyl homoserine lactones (AHLs) are used by over 50 Gram-negative bacteria species, including several pathogenic ones which are important threats to human health. AHLs backbone consist of three main sections, the homoserine lactone ring (biosynthesized from *S*-adenosylmethionine), the central amide group, and the side chain which is specific to each bacterial strain and can vary in length and level of oxygenation with a 3-oxo group (such as in *Vibrio fischeri* or in *Pseudomonas aeruginosa*) or a (3*R*)-hydroxyl functional group (such as in *Vibrio harveyi*) (Table 1). The alkyl chain with a length varying from 4 to 18 carbon atoms provides a hydrophobic character essential for diffusion through the cell membranes and binding within the hydrophobic pocket while the lactone, amide, and 3-oxygenated groups contribute to a strong hydrogen-bond network within the receptor binding site. In principle, different bacteria use different AHL signals [24]. However, some signals can be common to several species (such as *Vibrio fischeri* and *Erwinia carotovora*), affording a way for different bacteria to communicate between different species [25].

Chemically targeting AHLs can follow three strategies, either inhibiting their production by controlling the synthase, or decreasing their concentration in the extracellular environment (a process named quorum quenching) by chemical or enzymatic degradation [26,27,28] or by action of catalytic antibodies [29] or biodegradable molecularly imprinted polymers [30,31,32]. The third strategy is to interfere with the ligand-receptor interaction by adding antagonistic analogues that mimic natural ligands. Structural and biochemical data about the LuxR-type protein family have helped a lot to understand the ligand–protein interactions and accelerated the researches on QS modulation based on synthetic AHLs analogues [33,34,35,36]. In the following sections, we focus on how heterocyclic building blocks have been used as replacements or appendages in the three AHLs sub-sections. This can be as mimics of the lactone moiety, as a bioisostere group for the amide group, or as an additional pattern on the side chain. Other heterocyclic compounds with AHL-like QS activity are also described.

## 2. AHLs Structural Analogues Including a Heterocyclic Modification

### 2.1. Heterocycles as Replacement of the Lactone

**Figure 2:**
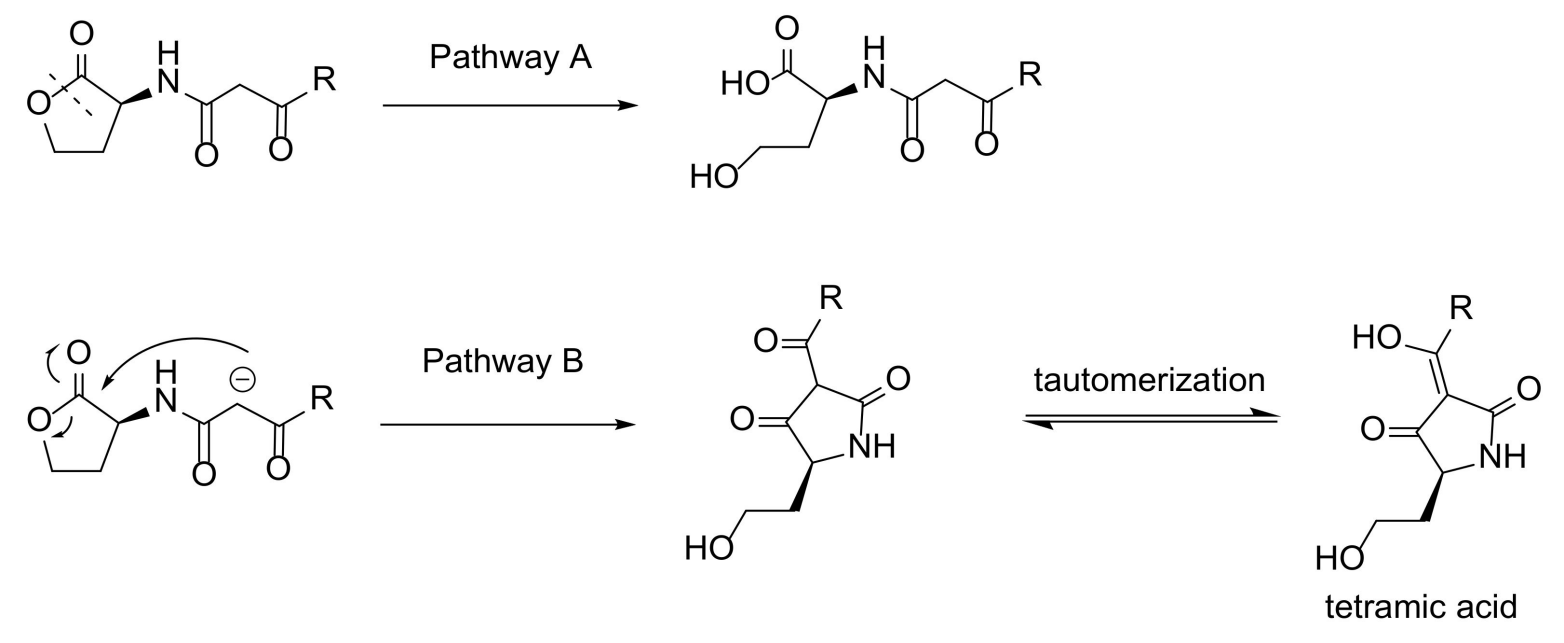
Degradation of 3-oxo-AHLs under basic conditions.

The lactone ring is crucial for interacting with the LuxR protein binding site. One strategy for designing QS modulators focusing on the lactone moiety of AHLs aims at keeping this key structural element intact by decreasing its ability to open up, or even making this opening impossible. Indeed, upon hydrolysis, the lactone opening product of AHLs shown in Figure 2 (pathway A) loses its activity for QS. However, intramolecular lactone opening hydrolysis can also occur, leading to a tetramic acid derivative (pathway B) which was identified as a potent antibacterial agent towards *P. aeruginosa* [28]. Several examples of AHL analogues in which the lactone has been replaced by a heterocyclic building block are listed below in this section. A second option, which relies on the use of scaffolds, which mimic the tetramic acid moiety, notably furanones and their analogues, are discussed in Section 3 of this review.

#### 2.1.1. Non-Aromatic Heterocycles as Lactone Mimics

**Figure 3:**
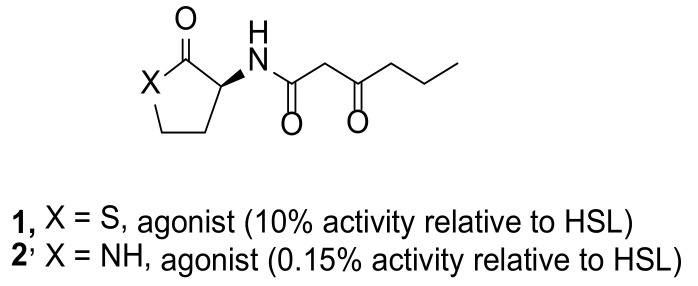
Thiolactone or lactam QS modulators in *E. carotovora* identified by Bycroft’s group.

The simplest analogs of the lactone are systems in which all but one atom are maintained in the backbone, such as in thiolactones or lactams. Many groups have reported such structurally close AHL analogs, among which several were found to be potent QS inhibitors by interacting with LuxR-type proteins. In 1993, Bycroft and colleagues [37] reported that thiolactones and lactams derived from native AHLs signals were able to restore the phenotype, which is involved in carbapenem biosynthesis in *E. carotovora* at a very low concentration (Figure 3).

**Scheme 1:**
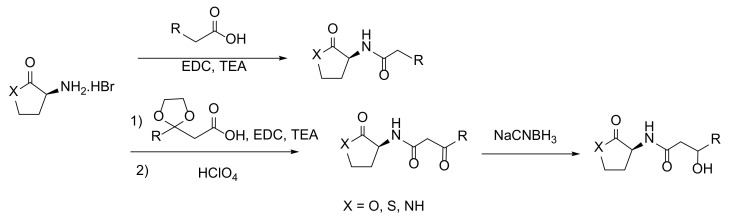
Access to thiolactone and lactam AHLs analogs.

The general synthetic route to access thiolactone or lactam AHLs analogs is shown in Scheme 1. The synthesis is based on the coupling of the thiolactone or lactam scaffold with various carboxylic acids potentially bearing a protected 3-oxo group in the presence of EDC. Deprotection and eventual reduction by sodium cyanoborohydride afforded the corresponding 3-oxo and the 3-OH AHL analogs, respectively. 

**Figure 4:**
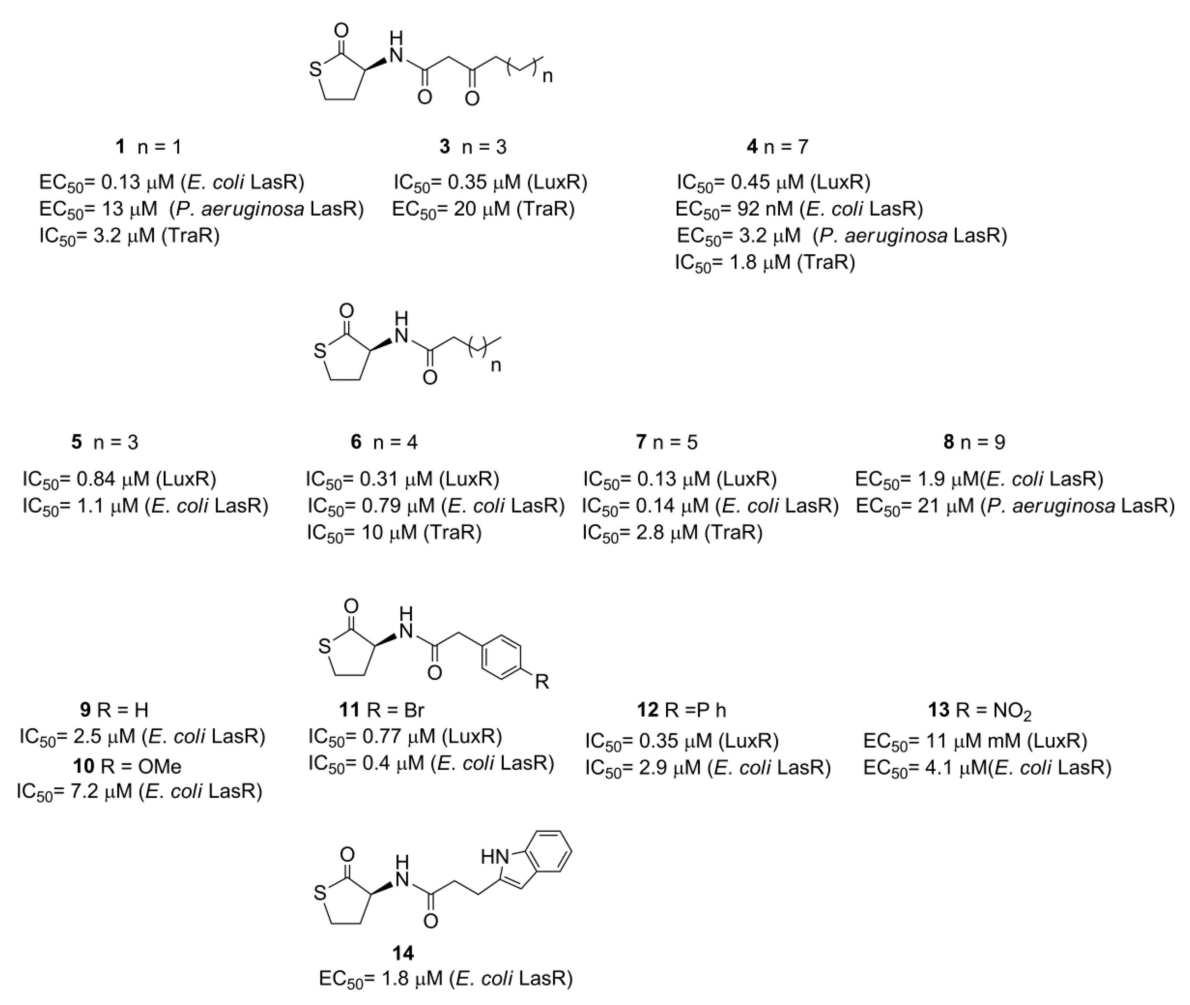
Thiolactone LuxR QS modulators identified by Blackwell’s group.

In 2011, Blackwell and her colleagues [38] examined a series of thiolactone analogues of AHLs with enhanced hydrolytic stability, with or without a 3-oxo moiety, or bearing a phenyl group in the side chain for evaluation of their QS activities. They discovered several highly potent QS agonists and antagonists of LuxR-type receptors (LuxR, LasR, TraR) in *V. fischeri, E. coli*, *P. aeruginosa*, *A. tumefaciens* (Figure 4). For example, thiolactones **1**, **3**–**7**, **9**–**12** displayed highly potent antagonistic activity on the LuxR-type receptor with IC_50_ values down to the nanomolar scale. Interestingly, the thiolactone **13** bearing a *p*-nitrophenyl group and thiolactone **14** bearing an indole motif in the side chain were found agonists whereas their corresponding AHL analogues are antagonists against *E. coli* LasR. Thus, the modification of the lactone part into thiolactone in highly similar structures to the native AHLs is not a simple mimicry, but also affects the activity in some cases.

**Scheme 2:**
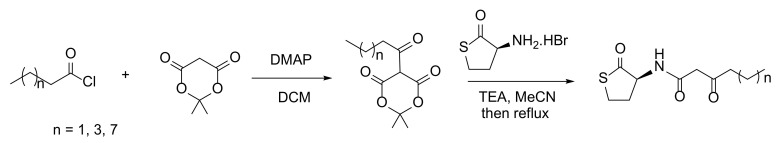
Synthetic approach towards acyl homocysteine thiolactones.

The synthetic scheme towards these thiolactones, described in Scheme 2, relies first on the formation of acylated Meldrum’s acid derivatives, which are then coupled with homocysteine thiolactone.

**Figure 5:**
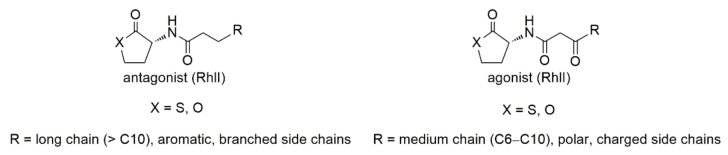
QS modulators for synthase RhlI protein in *P. aeruginosa*.

In addition to the activity versus the LuxR receptor, these acyl homocysteine d-thiolactones with a long chain (and corresponding acyl homoserine d-lactones) were found to inhibit the synthase RhlI in *Pseudomonas aeruginosa* (Figure 5) [39]. 

**Figure 6:**
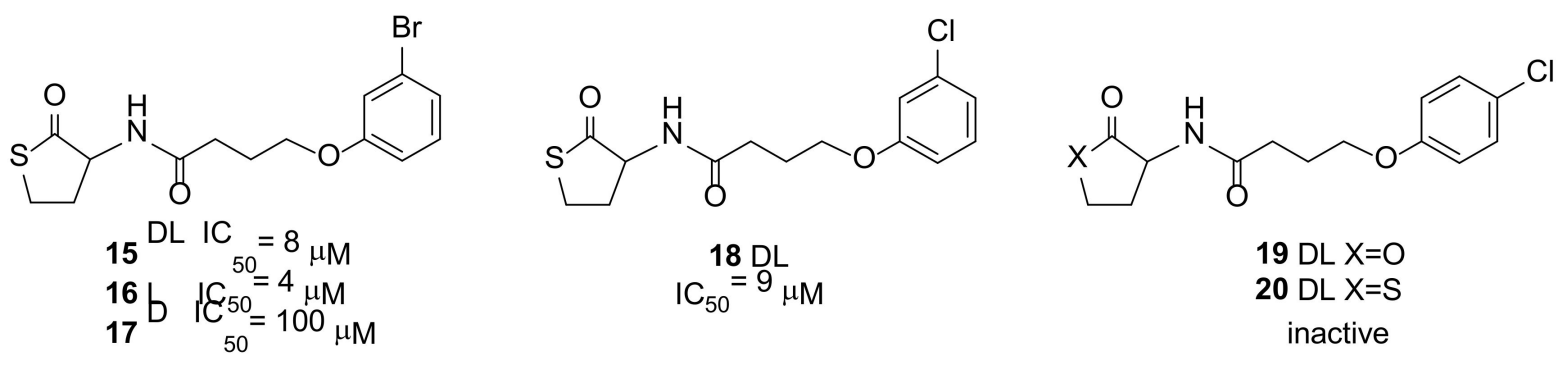
Thiolactone QS modulators of LuxR-type receptor from the Bassler’s group.

In *P. aeruginosa*, the protein RhlR receptor is a sensible target for designing chemical signals able to interfere with the QS pathway, either by agonism or antagonism. Here again, thiolactones have been considered. Bassler and coworkers [40] analyzed and identified *meta*-bromo-phenoxy thiolactones as potent inhibitors to block the production of the virulence factor pyocyanin and biofilm formation in *P. aeruginosa.* It was determined that RhlR, not LasR, was a relevant target though both of them were partially inhibited. In this work, thiolactones **15** and **16** were found more active than the others. No significant effect of the configuration (*S*/*R*) of the ligands on the biological activity was observed. Importantly, the most active molecule in this series, the *meta*-bromophenoxythiolactone **16** also protected *Caenorhabditis elegans* and human lung epithelial cells from being killed by *P. aeruginosa* (Figure 6).

**Figure 7:**
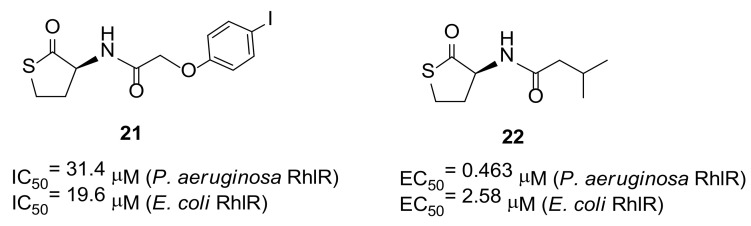
Thiolactone-based modulators targeting QS RhlR receptors from the Blackwell’s group.

In 2019, Blackwell and coworkers [41] reinvestigated their collection of acyl l-homocysteine thiolactones originally designed for targeting LuxR proteins, as non-native QS modulators of the activity of the RhlR receptor in *P. aeruginosa*. These compounds, more stable than lactones, proved to be very effective QS modulators (RhlR). The activities were also evaluated in the *E. coli* RhlR system. Compound **22** displayed agonistic activity in both *P. aeruginosa* and *E. coli*, but at 5-times lower concentration in *P. aeruginosa* compared to *E. coli*. These compounds exhibited high selectivity towards the RhlR receptor in *P. aeruginosa* relative to the QS LasR receptor (Figure 7).

**Scheme 3:**
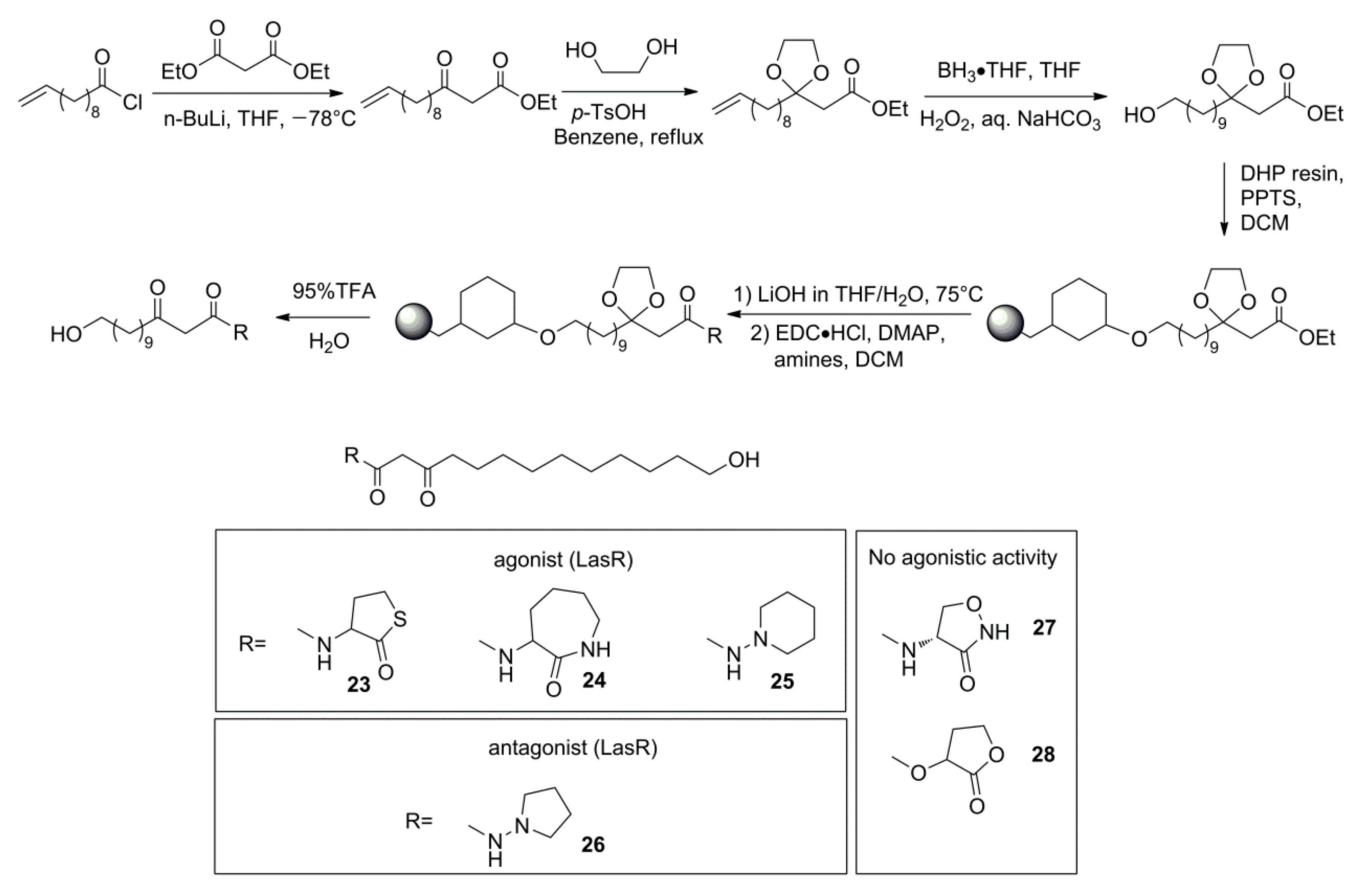
Examples of non-aromatic lactone-modified QS modulator identified by Suga’s group.

Building on their previous investigations on QS modulators, Suga’s group [42] synthesized and evaluated a series of AHL analogues in which the lactone is changed to various scaffolds. Several heterocyclic examples are included in this work, either with non-aromatic ones discussed below, and aromatic ones discussed in the next section. For their solid support synthesis, diethyl malonate was used as a nucleophile, which was coupled with an acyl chloride to generate the 3-oxo-propanoate moiety. After oxidation and attachment to a DHP resin, the ethyl ester was treated with LiOH to provide the acid which involved in a coupling reaction with various amines. The attached amide was then treated with TFA to be released as the final product with a hydroxyl group at the terminus of the acyl chain (Scheme 3). The thiolactone **23**, the lactam **24** and the piperidine derivative **25** were found agonists whereas the pyrrolidine derivative **26** was found to exert antagonistic activity. The amino-isoxazolidinone derivative **27** and the lactone **28** exert no agonistic activity.

**Scheme 4:**
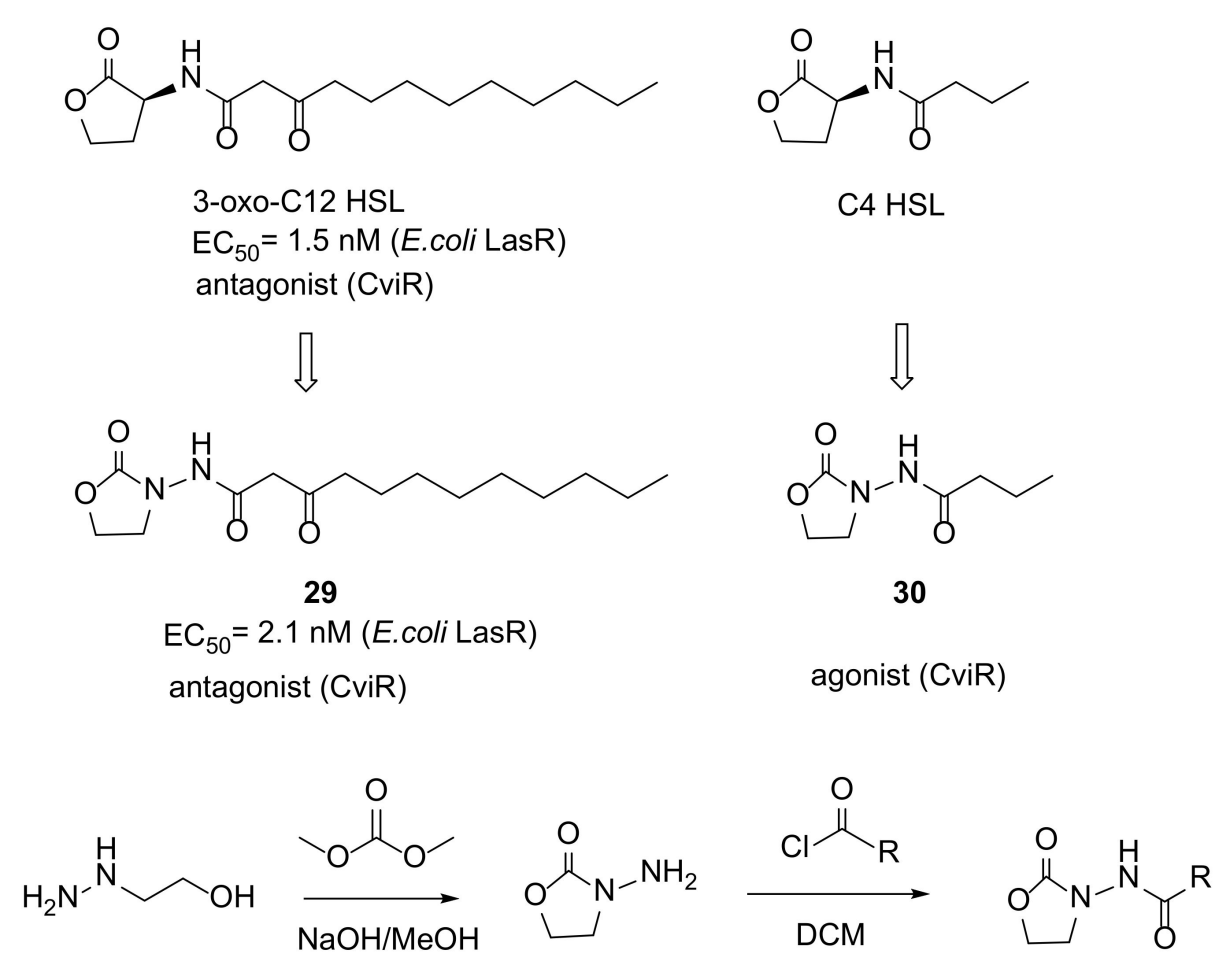
3-Aminooxazolidinone AHLs analogues.

Sintim and coworkers [43] revealed that oxazolidinone-based AHL analogue **29** was a very potent agonist which induced bioluminescence with remarkably similar intensities to the native ligand (3-oxo-C12 HSL) in the reporter strain *E. coli* pSB1075 containing QS-regulated bioluminescence genes. In another biological assay, the authors found that compounds **29** and **30** responded to the CviR QS system in *C. violaceum* CV026. Compound **30** was able to act as an agonist to activate the violacein production whereas compound **29** could inhibit this phenotype in high concentration. Regarding their synthesis, 3-aminooxazolidinones were prepared from the reaction of dimethyl carbonate with 2-hydrazinoethanol. Followed acylation with acyl chlorides gave the final oxazolidinone-based AHL analogues (Scheme 4).

#### 2.1.2. Aromatic Heterocycles as Lactone Mimics

As mentioned above, the part of QS modulators is at risk of being enzymatically degraded by lactonases. Thiolactone can undergo also some level of hydrolysis. To avoid this problem, some groups have considered the use of more stable scaffolds to replace the lactone head group for designing novel QS modulators, including heterocyclic aromatic rings discussed below.

**Scheme 5:**
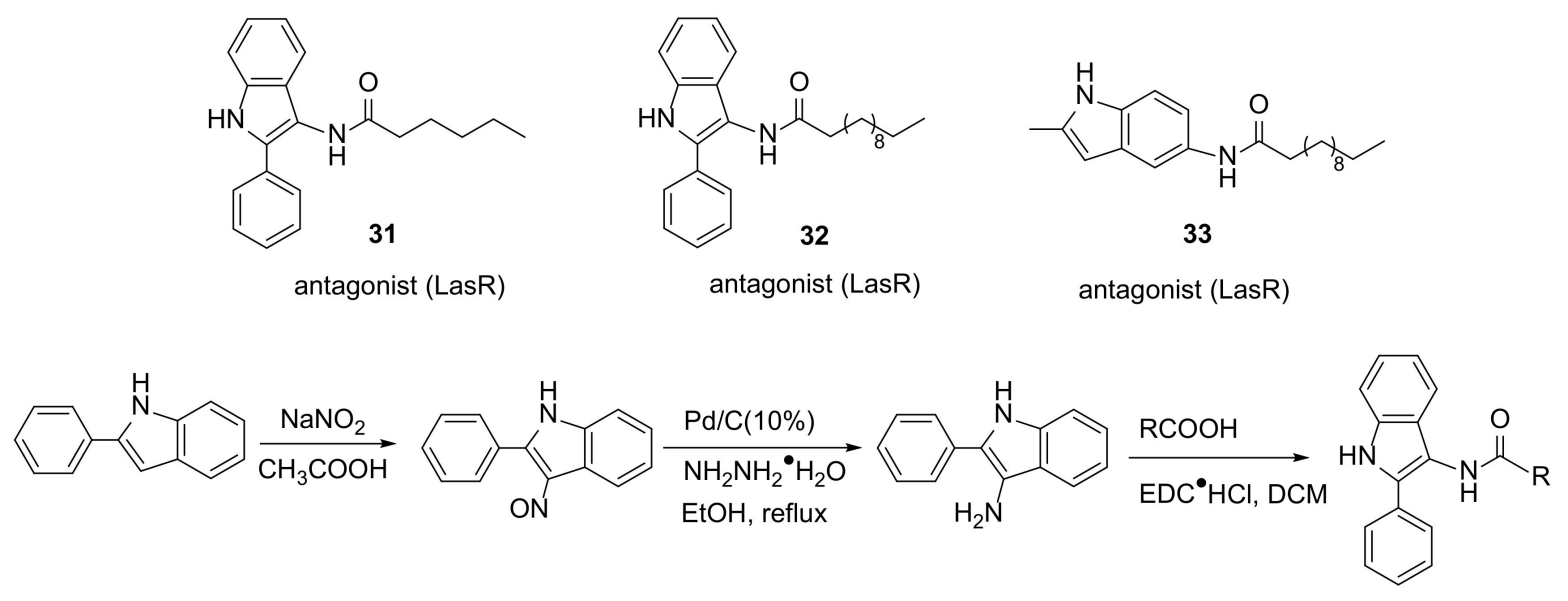
Heterocyclic aromatic replacement of AHLs lactone reported by Kumar’s group.

In 2015, Kumar and coworkers [44] replaced the lactone motif with an indole one and evaluated their QS inhibition by GFP fluorescence assays in *P. aeruginosa*. Compounds **31**–**33** displayed a 44% to 65% reduction of GFP fluorescence at 250 μM, among which antagonist **33** was the most active. In this work, the preparation of the indole-based AHL analogs involved in the coupling of carboxylic acids with substituted amino indoles. The substituted amino indoles were obtained from a two-steps procedure, including the nitrosation and the reduction of the nitroso intermediate with hydrazine in presence of palladium as catalyst (Scheme 5).

**Scheme 6:**
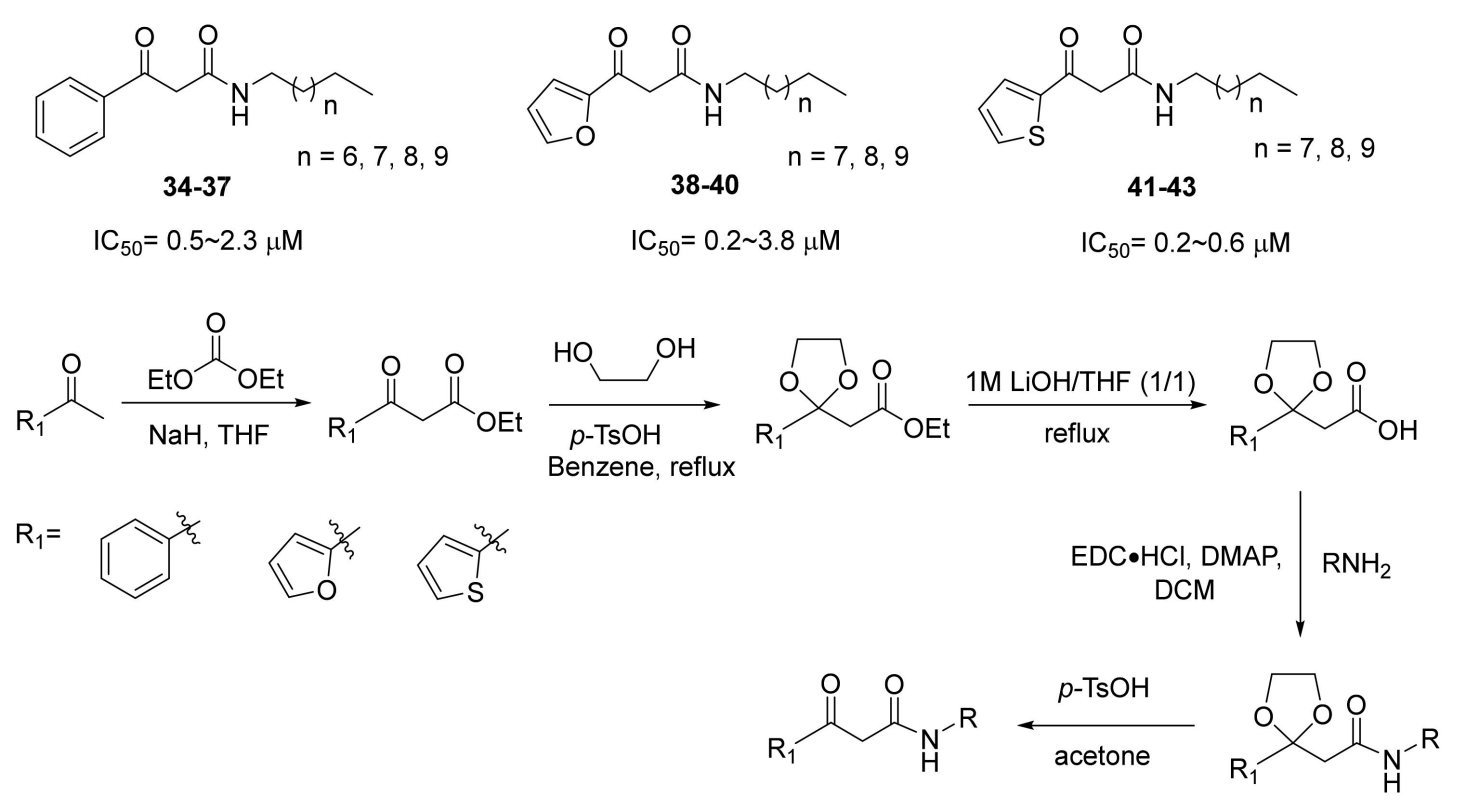
Furan and thiophene-containing LasR QS antagonists.

Blackwell and colleagues [45] reported also a series of AHL analogues in which the lactone is replaced by an aromatic moiety, some of them exhibiting very potent antagonistic activity against LasR reporter systems. On the basis of the phenyl derivative **34** discovered by a high-throughput screen of small chemical molecule libraries structural variations on the ring, notably using heterocyclic scaffolds, were performed for searching derivatives with improved potencies. Furans **38**–**40** and thiophenes **41**–**43** displayed similar antagonistic activities to the phenyl derivative **34**. The importance of alkyl chain length was noted, with side chains of 10 to 12 carbons giving similar activity as compound **34**, whereas analogues with shorter chain length (C5–C8) led to lower antagonistic activity, likely lacking from sufficient hydrophobic interaction with the receptors. On the other side, a stronger potency for heterocyclic analogues (**38**, **39**, **41**, **42**) than compound **34** in LasR was noticed. This series of analogues was obtained through the sequence shown in Scheme 6. The acylation reaction of acetophenones with diethyl carbonate gave the corresponding 3-oxo-propanoates, which were further protected as a ketal. Hydrolysis of the ester generated the carboxylic acid which was then coupled with various amines to yield the amides. Deprotection of the ketal using *p*-TsOH furnished the target products.

**Figure 8:**
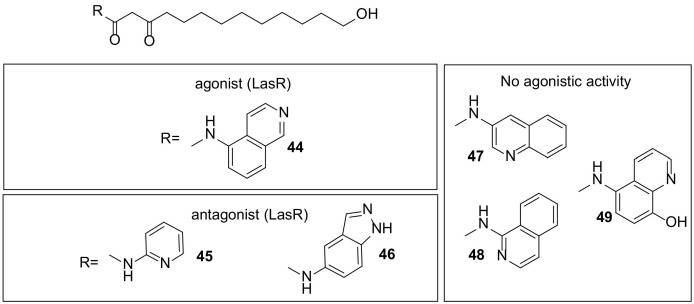
Examples of heteroaromatic QS modulator identified by Suga’s group.

Suga’s work already mentioned in the previous section [42] also included a set of heteroaromatic analogues (Figure 8), prepared by the same route shown in Scheme 3. These aromatic heterocyclic systems bear at least one nitrogen atom that provides H-bonding abilities for interactions within the receptor binding site. The evaluation as LasR antagonists of aminopyridine **45** and the indazole derivative **46** indicated that the position of the heteroatom plays a key role in the activities as well as for the isoquinoline derivative **44** for agonistic activity. The quinoline derivatives **47** and **49** and the isoquinoline **48** exert no agonistic activity.

**Scheme 7:**
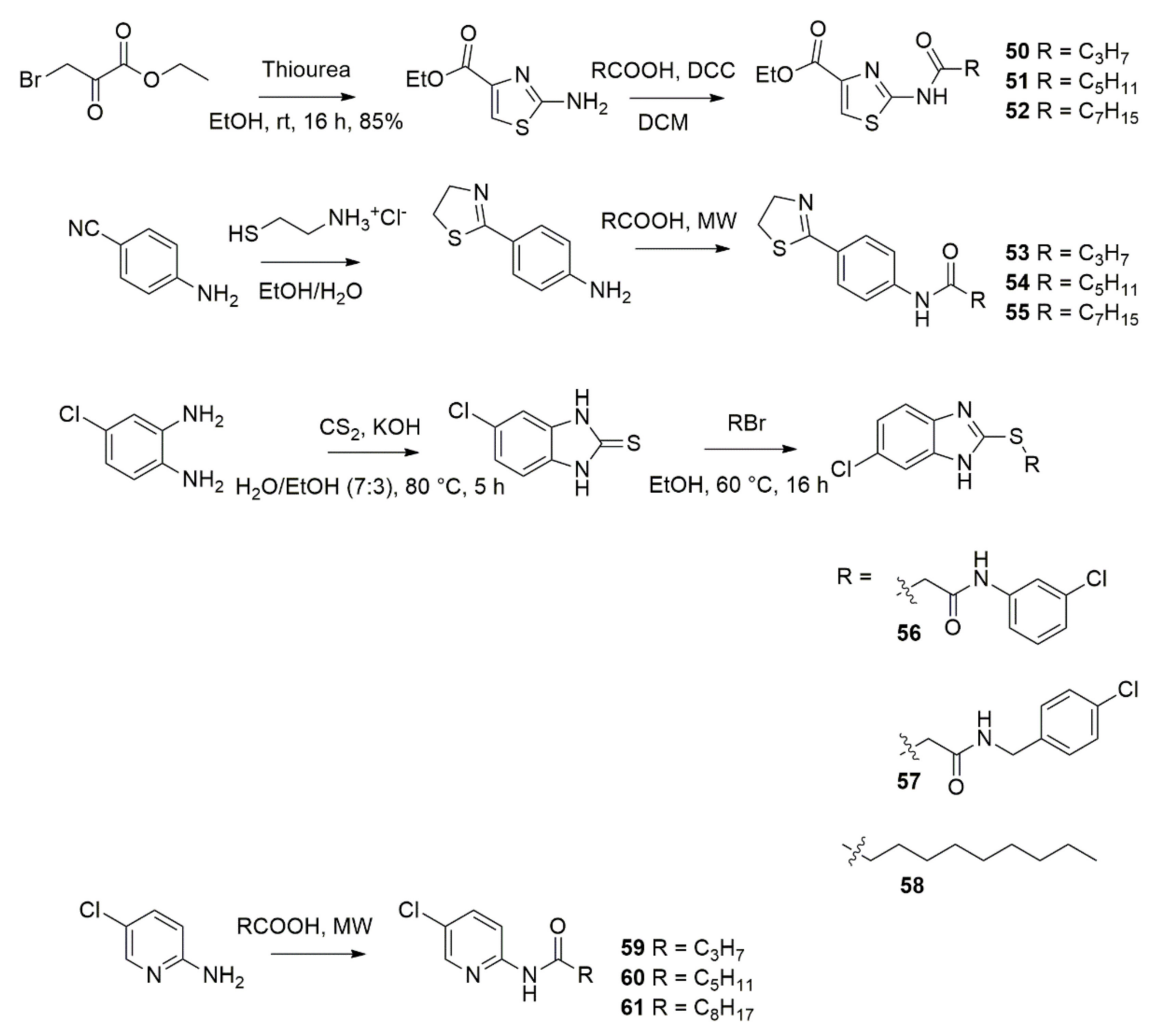
Synthesis of thiazoles, thiazolines, benzimidazoles, pyridines derivatives mimicking the AHL lactone moiety.

Other heterocyclic systems have been used for the design of AHL unrelated CviR-regulated QS modulators of such as thiazoles, thiazolines, benzimidazoles, pyridines represented in Scheme 7 [46]. The amino-thiazoles were prepared with thiourea and ethyl bromopyruvate and subsequently acylated using DCC as the coupling reagent. Thiazoline derivatives were synthesized by reaction of 4-aminobenzonitrile with cysteamine hydrochloride and subsequent acylation with carboxylic acid under microwave activation. The benzimidazoles derivatives were prepared by reaction of disulfide with 4-chloro-1,2-phenylenediamine to afford 5-chloro-benzimidazole-2-thione, which were further alkylated with various bromoacetamides or with bromononane. Finally, the pyridine derivatives were prepared from 5-chloro-2-aminopyridine through direct acylation with carboxylic acid under microwave activation. 

These compounds were evaluated for their ability to decrease the specific production of violacein in *Chromobacterium violaceum* CV026 in competition with C6-AHL. Thiazole **50** and thiazoline **53** with a C4 acyl chain were found to be active, as well as benzimidazole derivatives **56** and **57** with an amide functional group.

**Scheme 8:**
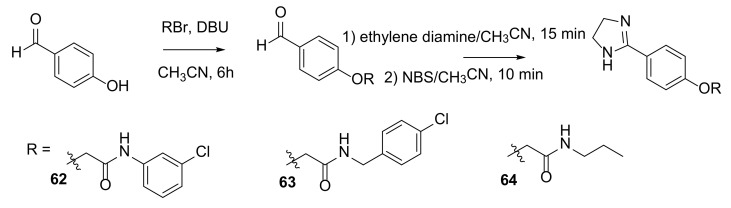
Synthesis of azoline derivatives.

The authors also reported azoline derivatives as a development of previous studies on other type of azoline derived compounds (Scheme 8) [47,48]. The synthesis involved the alkylation with DBU of 2-bromoacetamide derivatives obtained from the corresponding amines acylated with bromoacetyl bromide. The aldehydes were then subjected to a cyclisation step with ethylenediamine and subsequent oxidation with NBS under ultrasound irradiation. The 4-chlorobenzyl compound **63** inhibited 90% of violacein production at 100 µM whereas **62** and **64** exhibited 24% and 34% activity as QSI at the same concentration.

**Figure 9:**
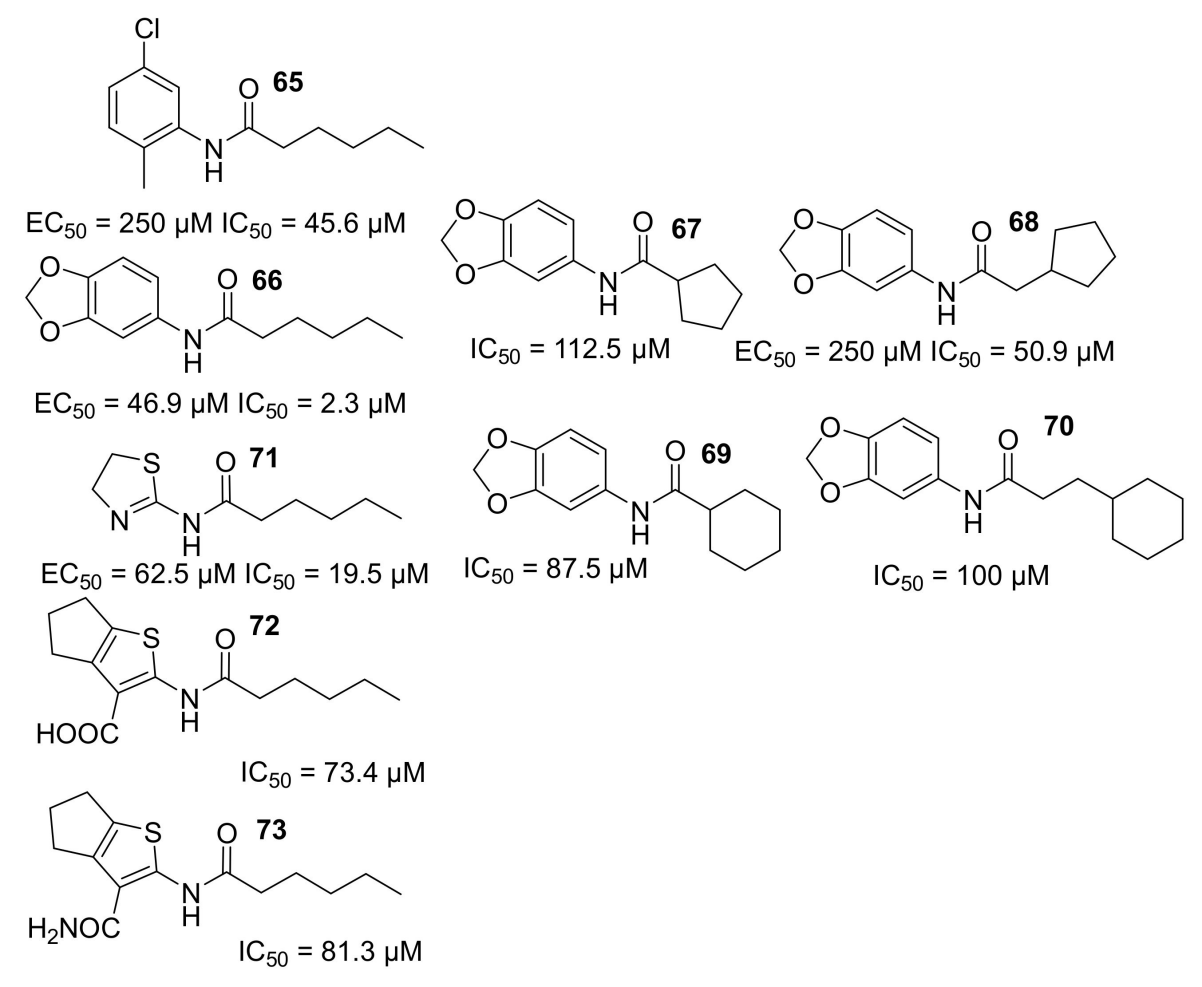
Structure of carboxamide derivatives as CviR modulators.

A recent work also described the modulation of CviR in *C. violaceum* CV026 using a small library of 19 compounds bearing the *N*-hexanamide non-polar radical or the *2H*-1,3-benzodioxole polar heterocyclic group [49]. Among all active compounds, the compound **66** (*N*-(1,3-benzodioxol-5-yl)hexanamide displayed the most interesting activity with an EC_50_ value of 46.9 µM and an IC_50_ value of 2.3 µM (Figure 9). The other compounds **65** and **67**–**73** displayed fair activity. Particularly, compound **71** was also interesting, with good activities for both agonistic and antagonistic. 

### 2.2. Heterocycles as Amide Bond Bioisosteres in AHLs

**Figure 10:**
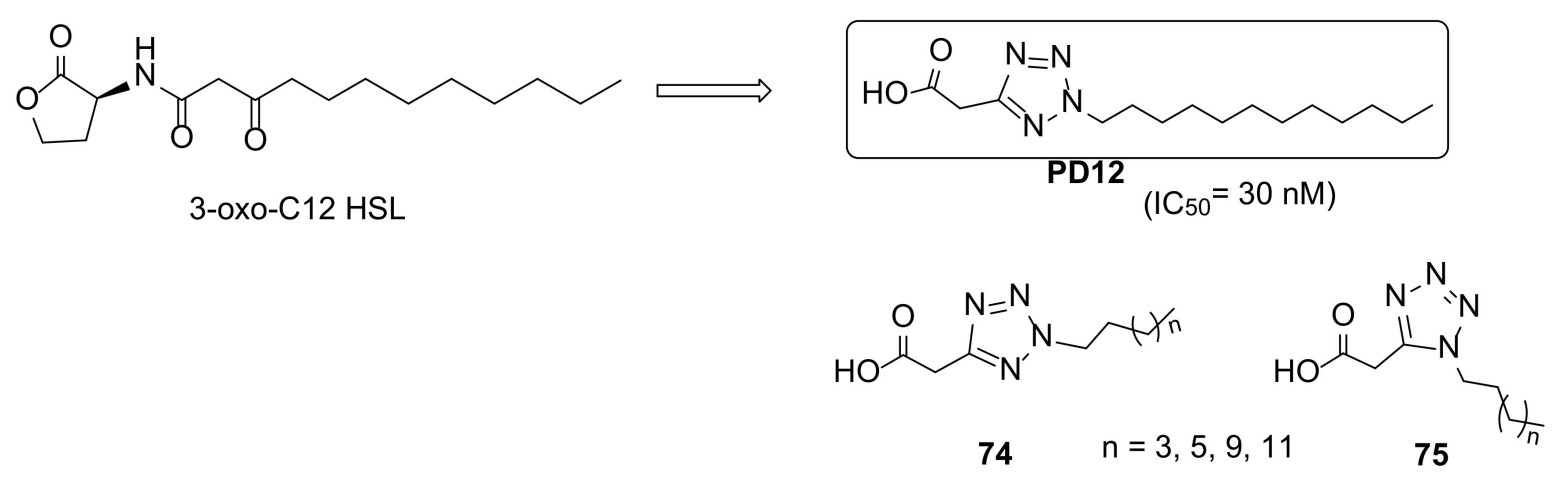
Tetrazole-containing QS inhibitor screened against LasR by Greenberg’s group.

In 2006, Greenberg’s group [50] reported the discovery of a 2,5-disubstituted tetrazole with a long alkyl chain named PD12 which was a highly potent inhibitor of LasR in *P. aeruginosa* with an IC_50_ value of 30 nM. The authors suggested that this inhibitor may bind to the specific region of LasR due to some structural similarity with the natural autoinducer, *N*-(3-oxo-dodecyl)homoserine lactone. In their study, 2,5-disubstituted and 1,5-disubstituted tetrazole derivatives **74** and 7**5** were designed, among which PD12 was identified as the most active. Overall, 2,5-disubstituted tetrazole compounds exerted higher inhibitory activity than 1,5-substituted ones (Figure 10).

**Scheme 9:**
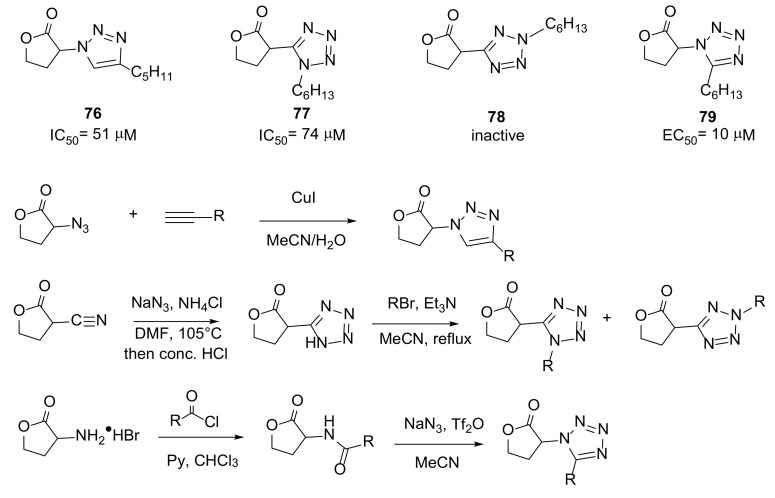
Triazole or tetrazole-based AHLs analogs reported by the Queneau and Soulère group.

Inspired by Greenberg’s group work showing the interest of the tetrazole scaffold, Queneau, Soulère, and coworkers described the synthesis and the biological evaluation on the LuxR-regulated QS of a series of triazole and tetrazole AHL analogues scaffolds (Scheme 9) [14]. Among the tetrazolic compounds, the lactone moiety was linked either to a carbon atom or a nitrogen atom of the tetrazole ring. It was found that the 1,5-disubstituted tetrazole compounds with a short chain were active. Interestingly, the analogue **77** and the analogue **79**, having the same chain length but differing only by the connection between the lactone and the tetrazole, either a C-C or a C-N bond, were found to exhibit inverse biological activity, respectively antagonist or agonist. The C-C connection between lactone moiety and the tetrazole in compound **77** is behaving more like an inverse amide, whereas C-N linkage in compound **79** can mimic the amide function. In contrast, compounds with substitution at the 2,5-positions of the tetrazole scaffold were completely inactive. The triazole derivatives such as compound **76** with fair antagonistic activity were synthesized via a Cu(I)-catalyzed azide-–alkyne cycloaddition reaction (CuAAC). The reaction of the cyano lactone with sodium azide gave the tetrazole, which was alkylated with alkyl bromides to generate two regioisomers. Alternatively, the coupling of acyl homoserine lactones with sodium azide led to the formation of tetrazolic compounds of type **79**. This latter compound was found to be a by accelerating the QS-regulated adherence of *A. ferrooxidans* cells on sulfur coupons, by triggering the QS system in planktonic cells and drive towards the sessile state, therefore to the formation of biofilms, through an effect on the expression of the *afeI* gene [51]. 

**Figure 11:**
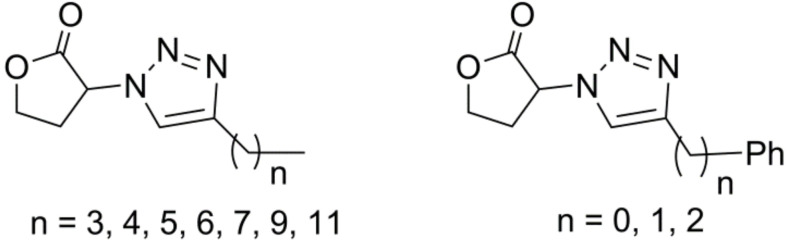
Triazole-containing QS modulator tested by Brackman’s group.

Independently, Brackman’s group [52] synthesized a set of AHL analogues in which the amide function was replaced by a triazole ring. These compounds were evaluated for their biological activity on QS in *E. coli*, *P. aeruginosa* and *B*. *cenocepacia*. They found that this family of triazole analogues exerted antagonistic activity in *E. coli* LuxR QS system. Only compounds bearing a long chain (C10 or C12) or phenyl group acted as antagonists (LasR) against the *P. aeruginosa* QSIS2 biosensor. In this study, the compound with a C12 alkyl chain was found to be the most active inhibitor, at the concentration of 1 mM, which resulted in a significant decrease in biofilm formation of the *P. aeruginosa* and *B*. *cenocepacia* strains. The synthetic route relied on the Cu(I)-catalyzed azide-alkyne cycloaddition reaction (CuAAC). Hansen et al. also described 1,4-disubstituted triazole derivatives as well as 1,5-disubstituted ones via Ru-catalyzed azide-alkyne cycloaddition reaction displaying moderate activities on LasR-regulated quorum sensing (Figure 11) [53].

**Scheme 10:**
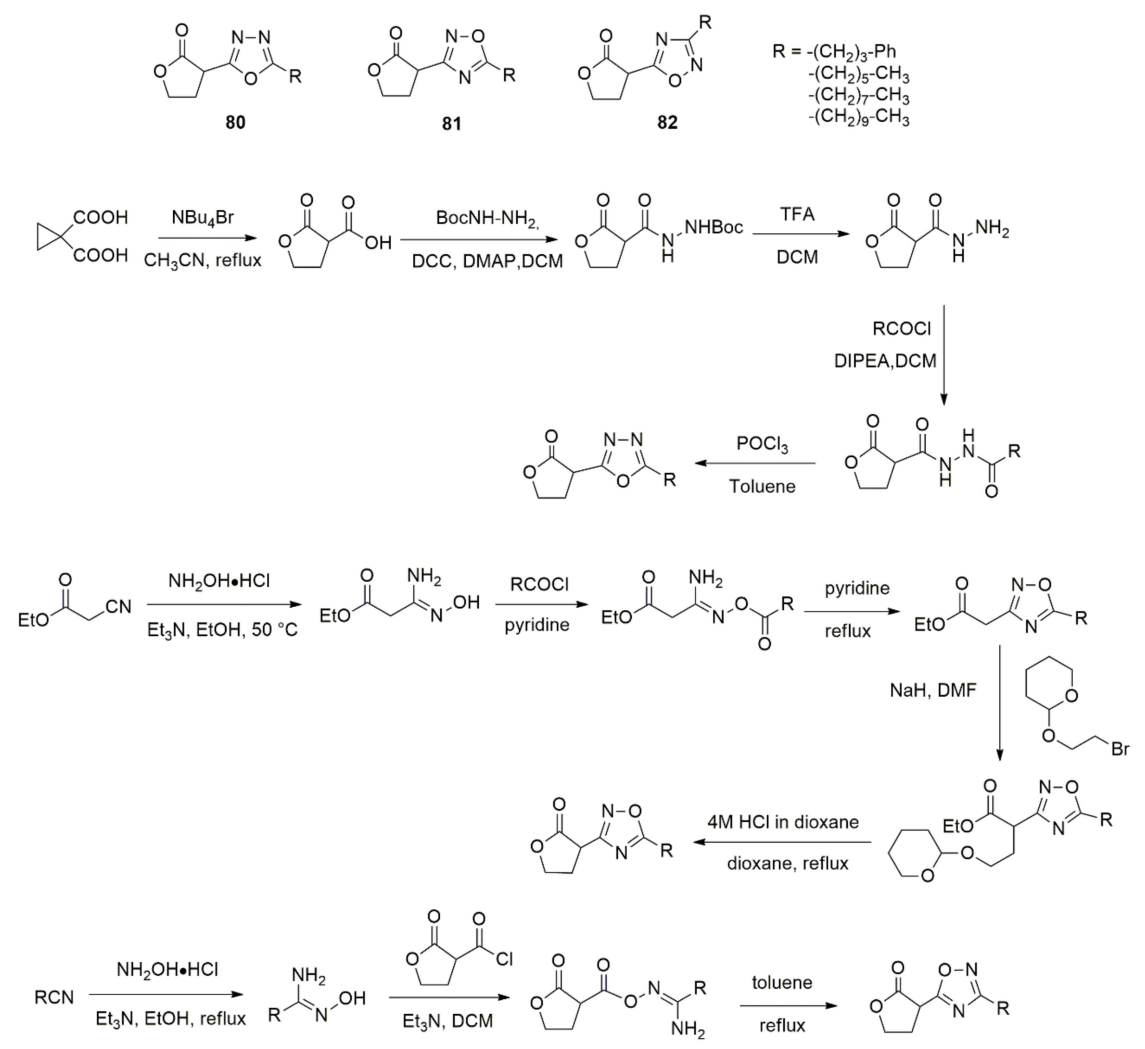
Oxadiazole-containing QS inhibitor tested by our group.

More recently, the Queneau and Soulère group investigated imidazole derivatives, some of them showing fair activity on LuxR-regulated QS [54]. Previously, the oxadiazole ring was also considered as replacement for the AHL amide, and a series of compounds were prepared, including 2,5-disubstituted 1,3,4-oxadiazole **80** and 3,5-disubstituted 1,2,4-oxadiazole (**81**,**82**) (Scheme 10). In this study, the lactone moiety was linked to the heterocyclic ring by a C-C bond. Three kinds of oxadiazole-based AHL analogs were obtained via three synthetic routes. For 2,5-disubstituted 1,3,4-oxadiazoles **79**, the lactone moiety was prepared from the rearrangement of 1,1-dicarbocyclopropane catalyzed by TBABr, followed by the coupling with hydrazine and linkage with corresponding acyl chloride. Dehydration using POCl_3_ led to the targeted 1,3,4-oxadiazole moiety. For 3,5-disubstituted 1,2,4-oxadiazoles **81**, hydroxylamine was used to convert the cyano group in ethyl cyanoacetate to the 3-amino-3-hydroxyimino derivative. After acylation and cyclization, ethyl 3-amino-3-(hydroxyimino)propanoate was converted to the 3,5-disubstituted 1,2,4-oxadiazole with an acetic group. The lactone was then constructed in the last sequence. Based on the same strategy, 3,5-disubstituted 1,2,4-oxadiazole **82** was synthesized from nitrile derivatives. Though none of these compounds were found to exhibit QS-modulation activity in the LuxR-type, they complete a very structurally diverse library of amide bioisosteres ready for being tested on other systems.

### 2.3. Heterocycles as Appendages Modifying the AHL Side Chain

**Scheme 11:**
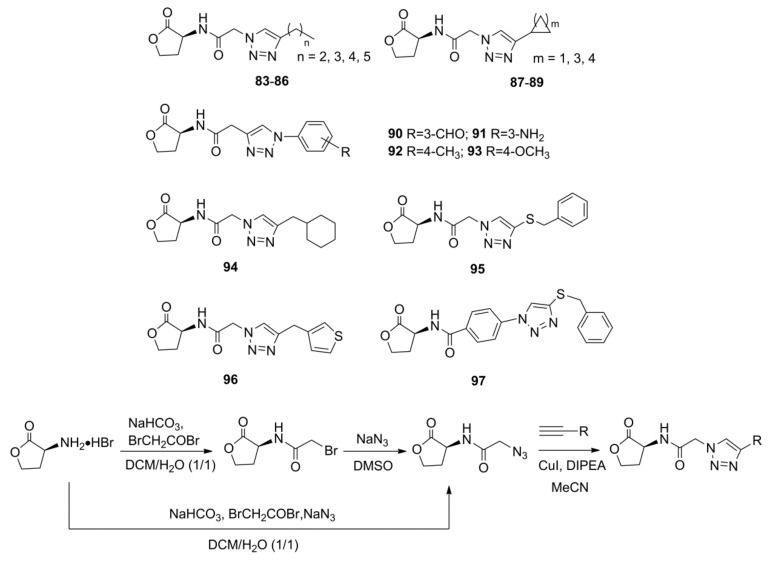
Triazole-containing QS modulator.

The type AHL acyl chain is a specification for each bacterial strain. It is a way for bacteria to compete against each other, long-chain AHLs being for example antagonists of the QS of bacteria having short-chain autoinducers. Apart from the chain length, several modifications can be envisaged, such as adding a bulky and or aromatic appendage. Among such analogs, several triazolyl systems have been reported, taking advantage of their straightforward construction by cycloadditions of alkynes with azides. Blackwell’s and Nielsen’s groups collaborated for reporting the design and biological screening of a large number of novel triazole derivatives targeting the LasR QS system in *P. aeruginosa* (Scheme 11) [55]. Several were found to potently inhibit the LasR receptor. In this series of triazole derivatives of different chain length (**83**–**96**), the nitrogen atom of the triazole ring provided a binding site with the LasR protein which mimicked the 3-oxo moiety of the natural ligand. Analogues with short chain (n = 3, 4) were stronger antagonists with an IC_50_ value up to 3.3 μM. For triazoles terminated with a cycloalkyl ring (**87**–**89**), compounds with larger rings (m = 3, 4) displayed higher antagonistic activity than smaller rings. Among all antagonists in their study, compound **97** bearing a thiobenzyl substituent was among the most active antagonists with an IC_50_ value of 2.6 μM. 

The activity of these compounds was evaluated with respect to the activity on the Las, Rhl, and Lux-regulated QS systems (in *E. coli*). For the LuxR pathway, compound **90** was found very active with an IC_50_ value of 5 μM [53] whereas compounds **83**, **87**, **88**, **96** displayed lower antagonistic activity. For the agonists, the other compounds activated the QS LasR system in *E. coli* with an EC_50_ value ranging from 1 μM to 71 μM. It is interesting to note that some compounds display both antagonistic and agonistic activity in different strains. The key intermediate azidoacetyl homoserine lactones were readily prepared from homoserine lactone, involving the bromoacetylation and substitution of the bromide with azide. In this sequence, the one-pot procedure led to the azidoacetyl homoserine lactone in good yield. Further CuACC reaction generated the desired triazole. As compared to compounds in which the AHL amide function itself was replaced by a triazole ring, stronger effects were found overall for this series of compounds constructed on this amidomethyl-triazole backbone. The thiolactone **14** bearing an indole motif in the side chain was also reported as QS modulators (see Figure 4 in Section 2.1.1 on non-aromatic heterocycles).

## 3. Non-AHL Related Heterocyclic Compounds with AHL-Like QS Activity

Besides designing close analogs of the AHL backbone as reviewed above, the discovery of QS modulators having AHL-like activities can get the inspiration from the structure of natural compound for which QS modulation activity was proved or suggested after biological screening. It can also be the result of the virtual screening of chemical libraries using models of the receptor binding site. Examples of heterocyclic systems belonging to these two categories are shown below. First, a focus is made on naturally occurring furanones produced by algae and their derivatives, as well as on similar, nitrogen containing, analogues. Both exhibit some structural analogy with the tetramic acid scaffold arising from the rearrangement involving the lactone opening (see Figure 2, Section 2.1). Then, heterocyclic compounds, such as imidazolium derivatives identified after virtual screening, are briefly discussed.

### 3.1. Furanone Type Analogues

Naturally occurring *5H*-furanones produced by the alga *Delisea pulchra* [56] are very interesting compounds due to their anti-QS and antibiofilm activity. For example, the natural (*Z*)-4-bromo-5(bromomethylene)-2(*5H*)-furanone-C30 has been extensively studied and recent studies showed that this compound is able to bind to LuxR type protein such as LasR and RhlR in *pseudomonas aeruginosa* [57]. In 2017, Fabio Bellina and co-workers described a state of the art of the chemistry of furanones and their relevant bioactivity [58]. Below we give an update on the furanone type compounds with examples reported since, and with compounds with similar structures.

**Scheme 12:**
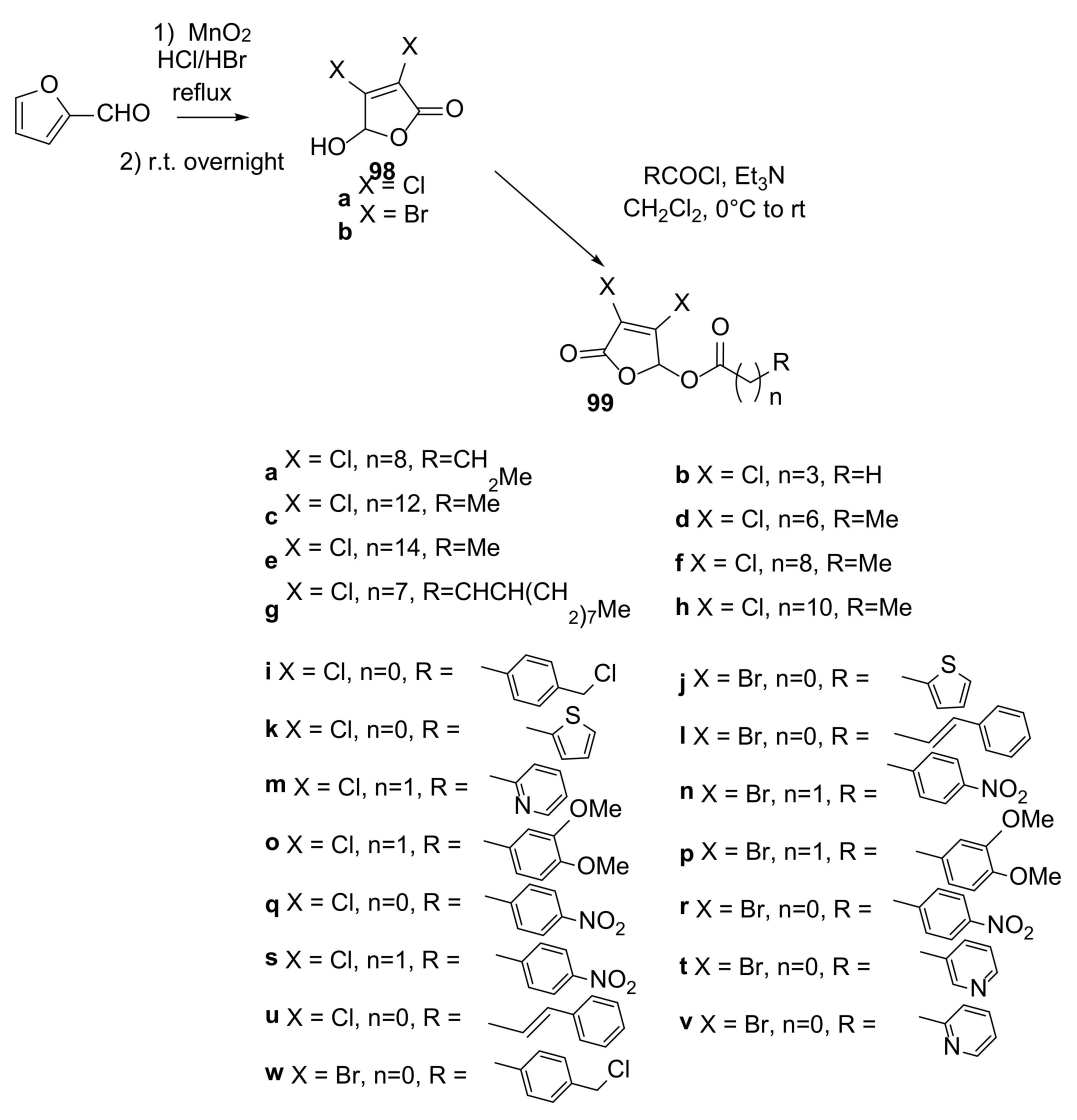
Synthesis of 5-hydroxyl-3,4-substituted-*5H*-furan-2-one derivatives.

More recent investigations have been reported in the literature about the synthesis and evaluation as LasR-QS inhibitors in *pseudomonas aeruginosa* of 5-hydroxyl-3,4-substituted-*5H*-furan-2-ones obtained from 2-furaldehyde (Scheme 12) [59]. Among all compounds, the dibromide **99p** was found to be very interesting with the most potent QSI and displaying also antibiofilm activity.

**Scheme 13:**
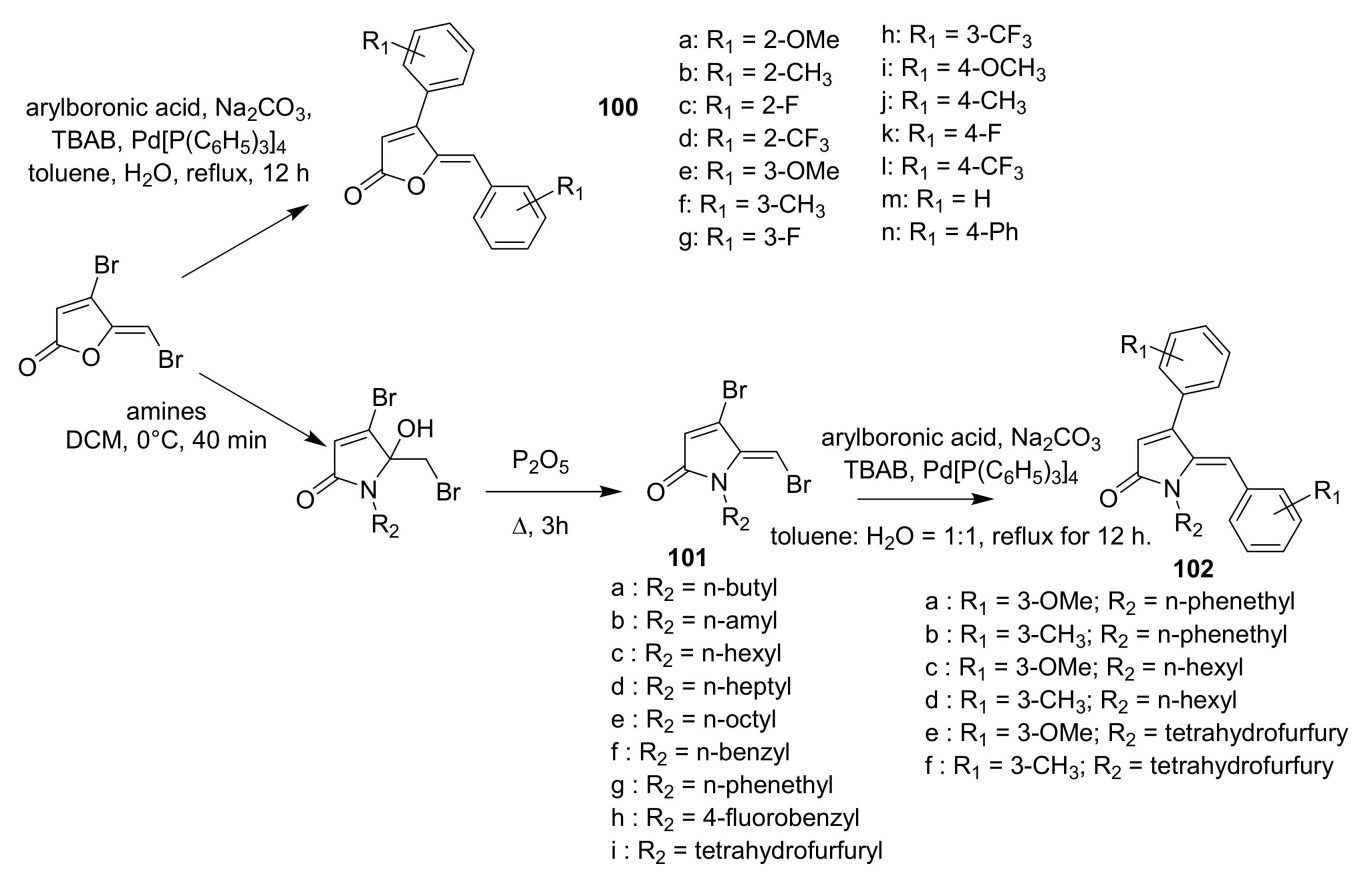
Synthesis of biaromatic furanone and aryl-substituted pyrrolidone derivatives.

In 2020, three series of biaromatic furanone and aryl-substituted pyrrolidone derivatives as LasR-dependent quorum sensing inhibitors have been synthesized starting from the furanone-C30 (Scheme 13) using a Suzuki–Miyaura cross-coupling as the key step [60]. The authors showed that the brominated pyrrolones with benzyl and n-hexyl substituents had significant quorum sensing inhibition activity. Compound **102b** is a potent quorum sensing inhibitor with antibiofilm activity in *P. aeruginosa*.

**Scheme 14:**
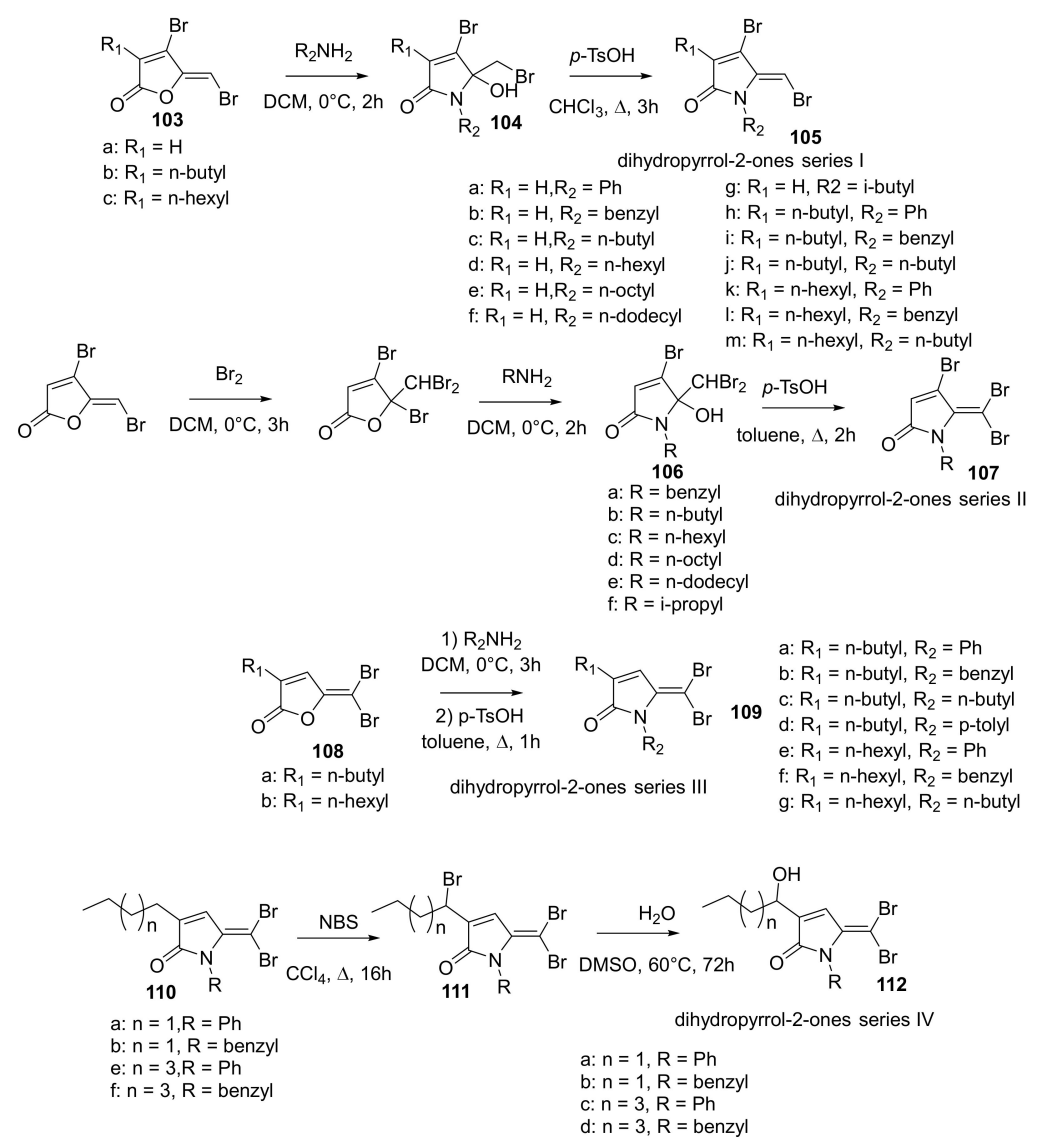
Synthesis of dihydropyrrol-2-ones derivatives as fimbrolide analogues.

In 2015, Naresh Kumar reported the synthesis and the study of natural fimbrolides analogues as LasR-regulated QS (Scheme 14). These analogues are structurally related to dihydropyrrol-2-ones and four series have been prepared [61]. The first series were synthesized starting from substituted (*Z*)-4-bromo-5(bromomethylene)-2(*5H*)- furanone-C30 in the presence of various amines to give the corresponding hydroxyl lactams, which were further dehydrated. The series II were prepared from (*Z*)-4-bromo-5(bromomethylene)-2(*5H*)-furanone-C30 via bromination, reactions with various amines followed by a dehydration reaction. The series III were prepared from 3-alkyl-5-dibromomethylene furanones with various amines and subsequent dehydration. Finally, some compounds from the series III were used to react with NBS to produce derivatives bearing α-bromo C3-alkyl chains, which were substituted in water to furnish α-hydroxyl C3-alkyl chains (series IV).

The authors tested their compounds as QSI and showed that natural fimbrolides from *D. pulchra* were more potent but the natural fimbrolides are cytotoxic to mammalian cell lines, while the dihydropyrrol-2-ones were found to be non-cytotoxic and hydrolytically more stable than fimbrolides.

The possibility that halogenofuranones undergo an addition-elimination pathway is likely a cause of toxicity. This is why we found it useful to also prepare some compounds lacking the activated halogenoalkene group. Interestingly, compounds, such as the non-brominated furanone shown in Figure 1, appropriately bearing a hydroxyalkyl group able to offer an interaction point for the binding within the receptor, showed quite high inhibition power [15].

**Scheme 15:**
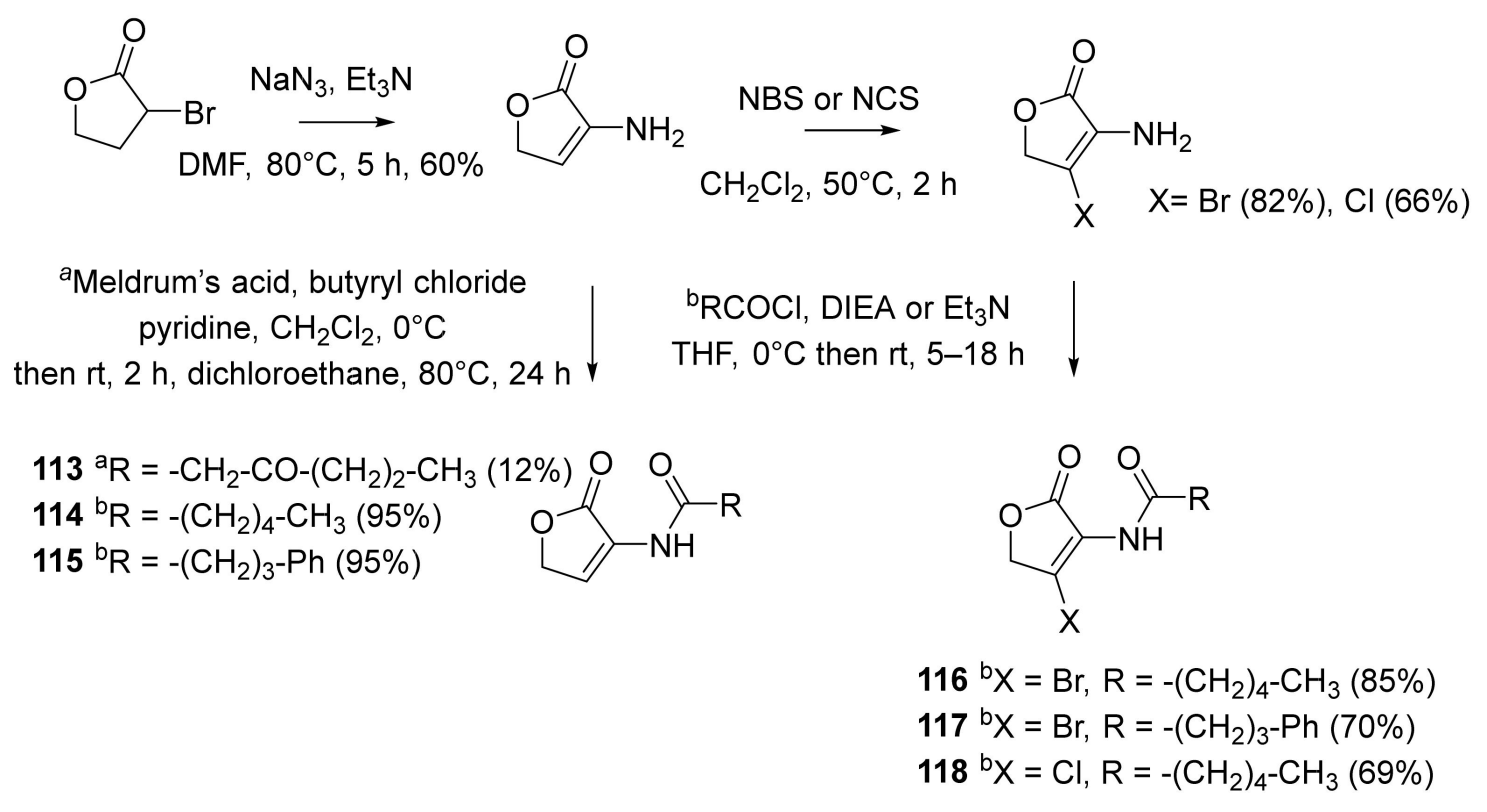
Synthesis of *N*-Acyl-3-amino-*5H*-furanone derivatives as mimics of AHLs.

Doutheau, Soulère, and colleagues developed in 2008 the synthesis of *N*-acyl-3-amino-*5H*-furanone derivatives as mimics of AHLs displaying inhibition of LuxR-dependent QS, with a hybrid design between the AHL and the furanone backbones [62]. These compounds were prepared from halogenated or hydrogenated enaminolactones, which were acylated in standard conditions (Scheme 15). These compounds exert activities with IC_50_ values ranging from 6.5–85 µM with the best activities for halogenated derivatives. 

### 3.2. Other Heterocyclic Systems

**Figure 12:**
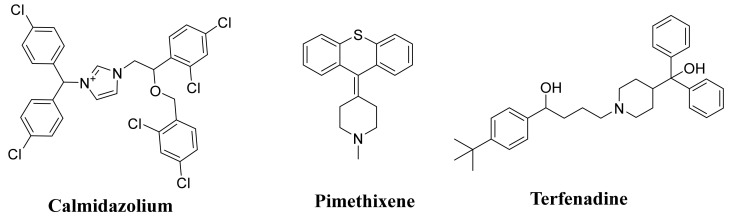
LuxR-regulated QS antagonists—calmidazolium, pimethixene, and terfenadine.

Other non-AHL-related heterocyclic compounds with AHL-like QS activity have been identified by our group through virtual screening targeting LuxR-type proteins. This study resulted in the identification of several LuxR-regulated QS antagonists namely calmidazolium (IC_50_ = 7 µM), pimethixene (IC_50_ = 56 µM), and terfenadine (IC_50_ = 92 µM), all three possessing an heterocyclic moiety (Figure 12) [63].

**Scheme 16:**
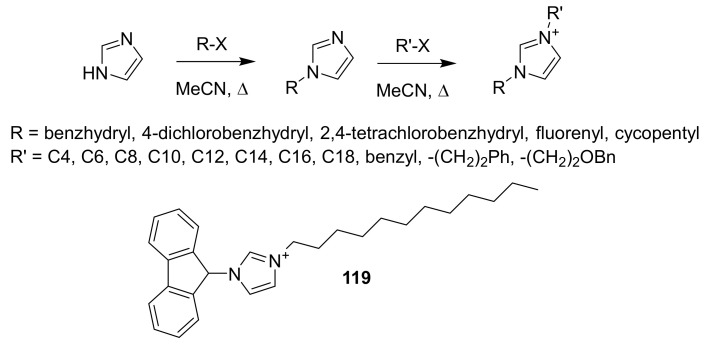
Synthesis of *N*,*N*′-alkylated imidazolium-derivatives.

This work was then extended to the synthesis and biological evaluation of simplified built on the imidazolium backbone found in calmidazolium, namely disubstituted imidazolium salts with benzhydryl, fluorenyl, or cyclopentyl substituent, and alkyl chains of various lengths. This investigation led to a fluorenyl and dodecyl disubstituted imidazolium **119** with an IC_50_ of 1.4 µM (Scheme 16) [64].

This type of compounds, *N*,*N*′-alkylated imidazolium-derivatives, such as compound **119**, also exert quorum sensing inhibitors targeting the *Pectobacterium atrosepticum*-induced symptoms on potato tubers [65].

## 4. Conclusions

This review highlights the usefulness of heterocyclic chemistry strategies in the design of analogues of acyl homoserine lactones (AHLs). Heterocyclic building blocks, aromatic or non-aromatic, have been used for replacing all three structural zones of AHLs, either the lactone ring, the central amide function, and the alkyl side chain. For the lactone ring replacement, the benefit is to limit its instability and the loss of activity upon hydrolysis. For the amide, it is to form a bioisostere group providing both the rigidity and intense H-bonding ability. For the side chain, it can be the mimicry of the 3-oxo or 3-hydroxy functional group, and/or developing a bulkier appendage exhibiting the hydrophobic character required for non-polar interactions in the binding site. In addition, analogs of naturally occurring furanones extending further the scope of structures. Overall, a wide range of heterocyclic AHL analogs have been thus designed and evaluated with respect to their ability to modulate QS. By highlighting this bridge between heterocyclic chemistry methodology and the design of novel QS-active molecules, it is hoped that heterocyclic chemists find in bacterial quorum sensing a novel field of applications to their methodologies, and echoingly, biologists and chemical biologists focusing on QS will consider novel building blocks for varying the molecular design of QS-active candidates.

## Figures and Tables

**Table 1:** Representative strains, phenotype, receptors, and structures in AHL-dependent QS.

Bacteria	AHL Signal	LuxI/R	QS Phenotype
*Vibrio fischeri*	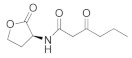 (OHHL)	LuxI/R	luminescence
*Vibrio harveyi*	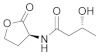 (3-OH-BHL)	LuxM/N	virulence
*Pseudomonas aeruginosa*	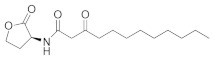 (OdDHL)	LasI/R; QscR	virulence, biofilm formation
*Agrobacteriumfabrum* C58	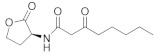 (OOHL)	TraI/R	Ti plasmid transfer
*Erwinia carotovora*	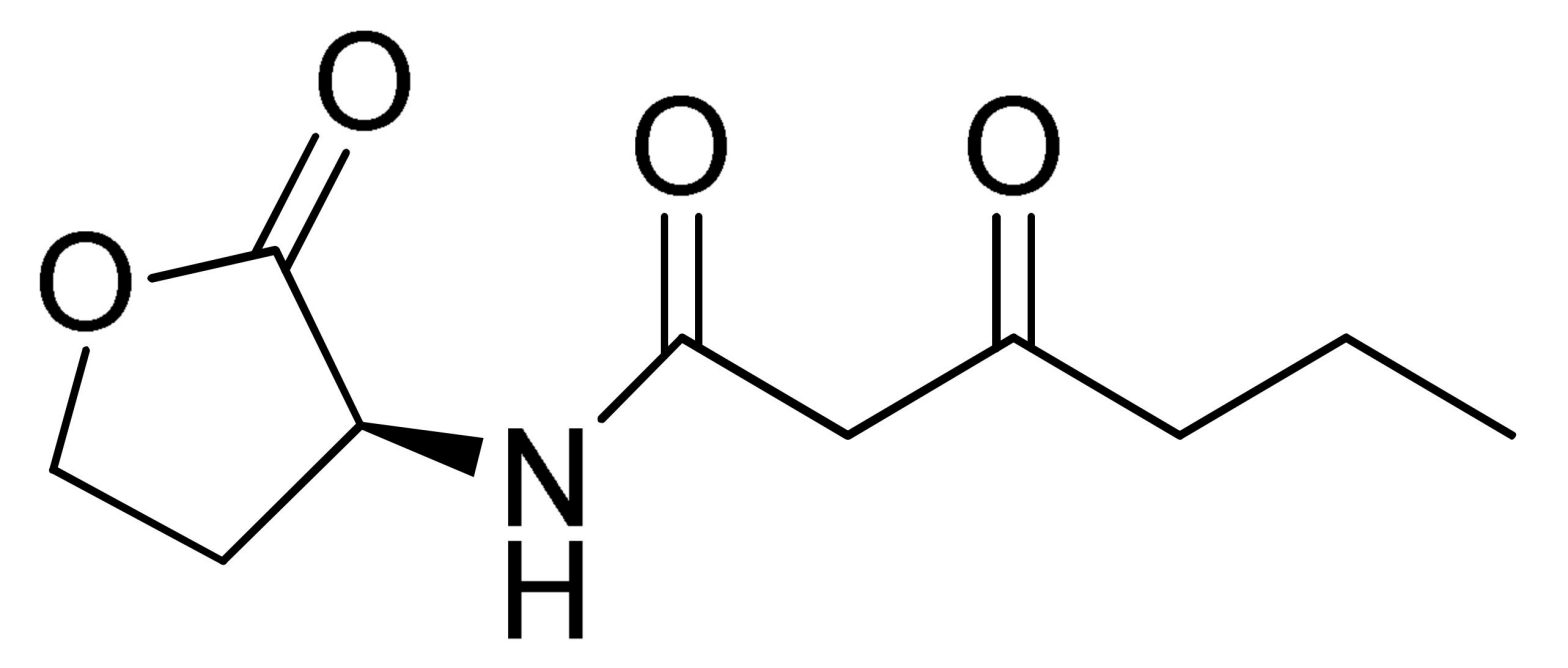 (OHHL)	ExpI/R; CarI/R	antibiotic production
*Serratia liquefaciens*	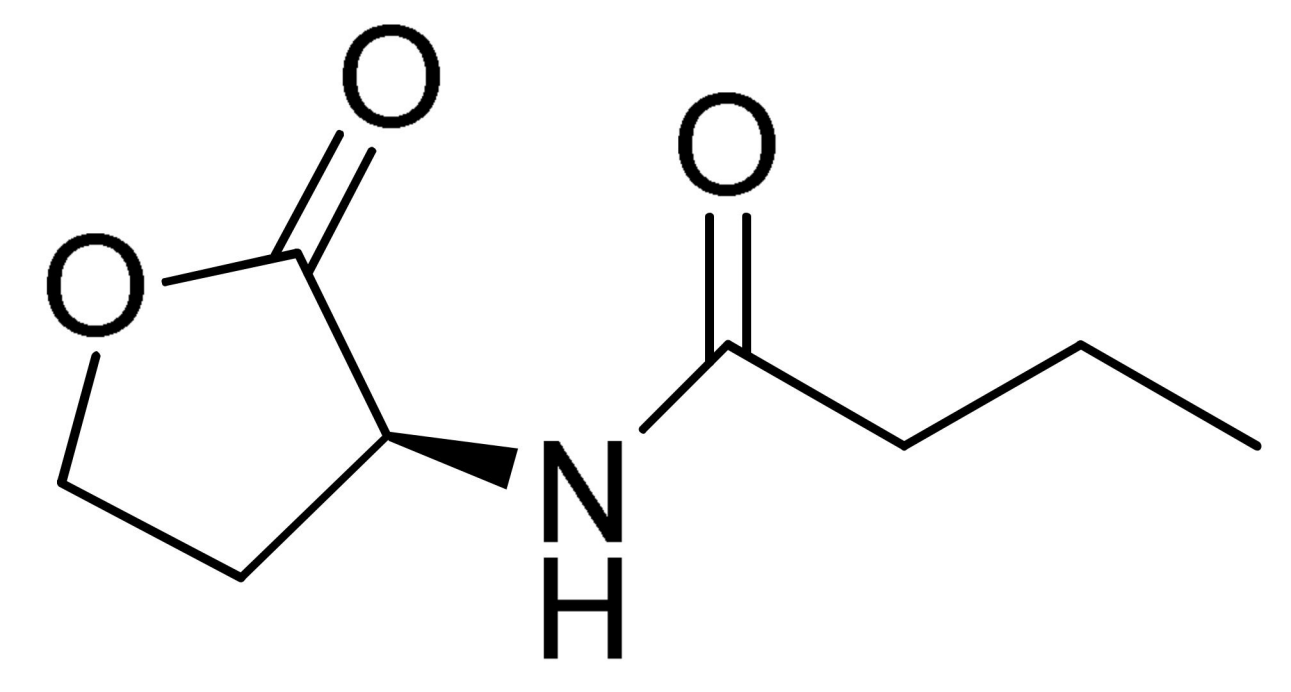 (BHL)	SwrI/R	antibiotic production
*Chromobacterium violaceum*	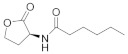 (HHL)	CviI/R	cyanide, pigment
